# Human Rights Violations among Men Who Have Sex with Men in Southern Africa: Comparisons between Legal Contexts

**DOI:** 10.1371/journal.pone.0147156

**Published:** 2016-01-14

**Authors:** Ryan Zahn, Ashley Grosso, Andrew Scheibe, Linda-Gail Bekker, Sosthenes Ketende, Friedel Dausab, Scholastica Iipinge, Chris Beyrer, Gift Trapance, Stefan Baral

**Affiliations:** 1 Department of Epidemiology, Johns Hopkins Bloomberg School of Public Health, Baltimore, MD, United States of America; 2 Institute of Infectious Disease and Molecular Medicine, Desmond Tutu HIV Centre, Cape Town, South Africa; 3 The Rainbow Project, Windhoek, Namibia; 4 School of Nursing and Public Health, University of Namibia, Windhoek, Namibia; 5 Center for the Development of People, Lilongwe, Malawi; University of Missouri-Kansas City, UNITED STATES

## Abstract

In 1994, South Africa approved a constitution providing freedom from discrimination based on sexual orientation. Other Southern African countries, including Botswana, Malawi, and Namibia, criminalize same-sex behavior. Men who have sex with men (MSM) have been shown to experience high levels of stigma and discrimination, increasing their vulnerability to negative health and other outcomes. This paper examines the relationship between criminalization of same-sex behavior and experiences of human rights abuses by MSM. It compares the extent to which MSM in peri-urban Cape Town experience human rights abuses with that of MSM in Gaborone, Botswana; Blantyre and Lilongwe, Malawi; and Windhoek, Namibia. In 2008, 737 MSM participated in a cross-sectional study using a structured survey collecting data regarding demographics, human rights, HIV status, and risk behavior. Participants accrued in each site were compared using bivariate and multivariate logistic regression. Encouragingly, the results indicate MSM in Cape Town were more likely to disclose their sexual orientation to family or healthcare workers and less likely to be blackmailed or feel afraid in their communities than MSM in Botswana, Malawi, or Namibia. However, South African MSM were not statistically significantly less likely experience a human rights abuse than their peers in cities in other study countries, showing that while legal protections may reduce experiences of certain abuses, legislative changes alone are insufficient for protecting MSM. A comprehensive approach with interventions at multiple levels in multiple sectors is needed to create the legal and social change necessary to address attitudes, discrimination, and violence affecting MSM.

## Introduction

Stigma and discrimination against men who have sex with men (MSM) have been documented worldwide. [1] High levels are associated with higher rates of negative mental health outcomes as well as unprotected anal intercourse, increasing risk of HIV and other STIs. [2–4] Hostile environments also impede building community, connectedness, and self-worth to protect against these outcomes. [5]

Thirty-eight of 54 African countries currently criminalize homosexuality. [6] In such environments, human rights violations have become increasingly visible over the past decade. [7, 8] Consistently, laws against same-sex activity are linked to blackmail and extortion against MSM, consisting of a threat to expose one's same-sex behaviour if payment of some kind cannot be paid. [9, 10] Criminalization also discourages MSM from accessing health services and decreases access to work, reducing affordability of certain needs. [11]

Malawi, Botswana, and Namibia are three countries with anti-gay laws while the South African constitution provides freedom from discrimination based on sexual orientation. While it is known that MSM in Southern Africa experience high rates of discrimination and abuse, this paper aims to explore the circumstances in which these MSM live, making inferences about how their experiences are informed by the legal context. [6, 12] We compare experiences of human rights abuses by MSM in Cape Town, South Africa with those of MSM in Gaborone, Botswana; Lilongwe and Blantyre, Malawi; and Windhoek, Namibia.

### Legal and Political Background

Complex interactions exist between sexual identities, behaviours, and orientations and between each of these and the legal framework, with MSM identifying as gay often being more vulnerable to abuse than those identifying as heterosexual. [13] African societies vary greatly in their tolerance of homosexuality. In many contexts, there is deep cultural and religious disapproval with large segments of the population holding religious beliefs that are deeply conservative, particularly when concerning sex and marriage. [14] This religious conservativism and cultural practices concerning sex, marriage, and children, intertwine with politics and increasing efforts to portray homosexuality as “unAfrican.” [14–16] Rather than protecting sexual minorities and addressing the needs of MSM, politicians and especially religious leaders across the continent have actively condemned MSM, calling for their persecution. [17] Political and other leaders use the denial of the existence of MSM in Africa and resort to anti-homosexual rhetoric when accused of corruption or mismanagement. [18, 19] Across the continent, leaders have equated homosexuals with external threats to manipulate misunderstanding and prejudice to achieve their own political gains. [12]

Anti-homosexuality laws also vary in their specificity and severity, with most criminalizing same-sex behaviour. [6, 8, 20] The most recent wave, some criminalizing the “promotion” of homosexuality, goes beyond criminalizing same-sex behaviour toward criminalizing same-sex identities themselves. [20] These laws criminalize not just the commission of an act, but the propensity to commit one. [21] Botswana and Malawi criminalize "carnal knowledge against the order of nature.” [8] In Namibia, homosexuality per se is not illegal, but anal sex between two males is considered illegal under common law sodomy provisions. [6]

Across the continent, penalties range from fines or detention to the death penalty. While long-term detention or death sentences are unusual, short-term arrests and extortion are common consequences of such laws. [22] In Namibia, the penalty is unclear, while in Botswana and Malawi, the penalty is imprisonment with a maximum of seven years in Botswana and 14 years in Malawi. [6, 23]

Where non-governmental organizations (NGOs) provide services for MSM, they face prosecution for "promotion" of homosexuality. [24] In Nigeria, a new law punishes registering, operating, or participating in gay organizations, and Uganda’s Anti-Homosexuality Act assented to in 2014 and overturned by the Constitutional Court six months later, included imprisonment for reaching out to homosexuals. [25, 26] Furthermore, criminalizing same-sex behaviour makes it difficult for donor agencies to commit to funding programs for MSM and for researchers to study them. [27] As such, it is difficult for NGOs to promote safer sex or conduct programs involving outreach and mass media, with serious consequences for HIV epidemics. [17]

In 1994, South Africa approved the new constitution promising to end discrimination on all grounds, including sexual orientation. [28] Though this legal victory did not necessarily reflect the attitude of many South Africans at the time, and may even have resulted in a homophobic backlash, it showed the marked post-apartheid excitement in the discourse of equality. The South African government committed in the word of law to acknowledge and uphold the human rights of gay, lesbian, bisexual, and transgender residents and citizens. [7, 29, 30].

## Methods

### Sample and recruitment

The methods for the study from which these data were obtained have been described elsewhere. [10, 31] The study was completed in peri-urban Cape Town, South Africa; Gaborone, Botswana; Windhoek, Namibia; and Blantyre and Lilongwe, Malawi. Eligible participants were at least 18 years of age, born male, reported anal intercourse with another man, were residents of the study cities, and were able to give oral consent in English or a local language. The study was anonymous and confidential, and with approval from the institutional review board, no written communications, including verbal consent scripts, were shared with participants to minimize risk of disclosing the participant’s sexual orientation or of participation in the study. Consent was documented by signature of the interviewer on the study documents.

In total, 737 MSM participated. In Botswana, Malawi, and Namibia, given the lack of gay venues, recruitment was done through snowball sampling by a community-based organization (CBO) in each country. The CBO in each country was chosen because of their previous experience working with MSM in their communities, and their capacity to conduct the study procedures. In total, 218 participants were recruited in Namibia and 202 in Malawi. In Botswana, 117 MSM were recruited because of difficulty in accessing this population and delays in local approval processes.

In South Africa, 200 MSM were recruited using venue-based sampling with peer referral at each venue, as previously described. [32] Recruitment staff visited bars, clubs, street locations, and social organizations, and approached men to inform them of the study and assess eligibility. Participants were asked to refer acquaintances also at the venue at that time. All potential participants meeting inclusion criteria were asked if they consented and offered the opportunity to participate.

### Survey instrument

A structured 45-question survey was developed through iterative rounds of input from a panel of experts in determinants of health, human rights, and HIV epidemiology (Survey Instrument in S1 File). The same instrument was piloted with MSM CBO members in each country, revised, and locally adapted. The final survey instrument used in each country included the same 45 questions. A CBO member in each city administered the survey, which took 25 minutes to complete, collecting information regarding demographics, rights abuses, HIV status, sexuality, HIV knowledge, perceived and experienced stigma relating to sexual orientation, access to health care, experienced discrimination, and sexual risk behaviour. No identifying information was collected and surveys were labelled with alphanumeric codes to allow anonymous linking of survey results with HIV-1/2 test results.

### HIV testing

Saliva samples were obtained from consenting participants to assess HIV-1/2 infection and analysed with OraSure OraQuick® rapid HIV-1/2 antibody test kit (Orasure Technologies, Bethlehem, PA, USA). This screening was for study purposes only and not for confirmatory diagnosis of HIV infection. All participants were informed of this during the consent process. Counselling for the value of HIV testing was provided to all participants, who were referred to appropriate local venues for diagnostic testing and counselling.

### Statistical analysis

Data analysis was performed using Stata 12 statistical analysis software. [33] Participants were considered to ever have experienced a human rights abuse if they had ever, because of their sexuality, been denied housing or healthcare, been blackmailed, beaten by the police, or raped. Preliminary analyses, including analysis of variance (ANOVA) and Pearson χ^2^ tests, tested for differences across study locations in sociodemographic characteristics and experiences of rights abuses. Bivariate logistic regression was used to model the relationship between each abuse and predictor variables.

Multivariate logistic regression was used to model the relationship between each abuse and study location after controlling for confounders. Each abuse was modelled separately as an outcome variable. Selection of predictor variables was based on available literature and findings from bivariate analyses. The final model was chosen by examining Akaike Information Criteria (AIC), a measure of statistical model quality. [34] Final predictors included country, age, education level, and employment status and whether the participant was originally from the study country. Other predictors included HIV status, sexual orientation, disclosure of orientation to family or healthcare workers, use of injection drugs, history of arrest, engagement in transactional sex, and number of male sexual partners in the previous six months.

### Ethical considerations

The studies were approved by the Institutional Review Board of the Johns Hopkins Bloomberg School of Public Health, the Research Ethics Committee of the University of Cape Town, the University of Namibia Institutional Review Board, and the Ministry of Health of Botswana. In Malawi, the local CBO, CEDEP, employed a previously-described internal review mechanism and approved the study. [10]

### Limitations

The cross-sectional design and lack of baseline data prevent any characterization of causality between associations described here and rights abuses in any country context. There may have been differences in interpretation of certain rights issues because of differences in language or other factors. A survey instrument relying on participant understandings of the term *rape* as was used in this study rather than behavioural measures may underestimate its prevalence. [35] Additionally, these are convenience samples generated by chain-referral and venue-based techniques. Those recruited using a venue-based approach may be more visible both because they are more open about their sexuality and because they are visiting venues known to be gay-friendly, possibly increasing rates of abuse. [13] Consequently, the differences observed in study populations across countries should not be generalized to all MSM within that country.

As race data were not collected, controlling for race was not possible. However, in South Africa, black and coloured individuals experience higher rates of discrimination regardless of sexual identity. [36] This suggests the rates of abuse reported here may overestimate the expected rates for white MSM and underestimate those expected for black or coloured MSM. As sample sizes were originally calculated to investigate HIV prevalence and risk among MSM at each site, the sample sizes are modest, affecting statistical power to detect true differences between groups. These issues will be addressed by larger studies where feasible.

## Results

### Sociodemographics and practices of study participants

Overall, participants were young, with mean ages between 24 and 26 years (Table 1). The majority had at least a secondary school education, and half were employed. The site with the greatest proportion self-identifying as gay, at 77.0%, was Cape Town. More identified as heterosexual in Malawi and Namibia, and these differences were statistically significant (p<0.01). A higher proportion, 68.5%, in Cape Town had disclosed their sexual orientation to a family member, compared to 60.3% in Gaborone, 44.5% in Windhoek, and 17.0% in Blantyre and Lilongwe (p<0.01). The pattern was similar for having disclosed sexual orientation to a healthcare worker.

**Table 1:** Selected Characteristics of Sampled MSM Overall and by Country.

Descriptive variables		Overall % (n/N)	South Africa % (n/N)	Botswana % (n/N)	Malawi % (n/N)	Namibia % (n/N)	p-value
**Age (mean yrs (SD))**		25 (5.80)	26 (6.87))	25 (4.78)	26 (5.31)	24 (5.51)	<0.01
**Born in country of survey**		93.34 (687/735)	97.50 (195/200)	82.91 (97/117)	92.54 (186/201)	95.87 (209/218)	<0.01
**Education**	No formal education	1.22 (9/735)	0.50 (1/200)	0.85 (1/117)	0.50 (1/201)	2.75 (6/218)	<0.01
	Primary	6.79 (50/735)	9.50 (19/200)	0.85 (1/117)	7.46 (15/201)	6.88 (15/218)	
	Secondary	54.48 (401/735)	46.00 (92/200)	36.75 (43/117)	51.24 (103/201)	74.77 (163/218)	
	Tertiary or Vocational	37.50 (276/735)	44.00 (88/200)	61.54 (72/117)	40.80 (82/201)	15.60 (34/218)	
**Employment**	Employed	50.61 (371/732)	60.30 (120/199)	49.14 (57/116)	51.24 (103/201)	41.94 (91/217)	<0.03
**Sexual orientation**	Heterosexual/straight	8.33 (61/732)	1.00 (2/200)	3.42 (4/117)	6.50 (13/200)	19.44 (42/216)	<0.01
	Homosexual/gay	57.03 (418/732)	77.00 (154/200)	66.67 (78/117)	40.50 (81/200)	48.61 (105/216)	
	Bisexual	32.61 (239/732)	18.00 (36/200)	29.06 (34/117)	53.00 (106/200)	29.17 (63/216)	
	Transgender	2.05 (15/732)	4.00 (8/200)	0.85 (1/117)	0	2.78 (6/216)	
**Disclosed sexual orientation to:**	Family member	46.05 (338/733)	68.50 (137/200)	60.34 (70/116)	17.00 (34/200)	44.50 (97/218)	<0.01
	Healthcare worker	26.26 (193/734)	50.00 (100/200)	24.14 (28/116)	8.96 (18/201)	21.56 (47/218)	<0.01
	Family or healthcare worker	51.50 (378/733)	76.50 (153/200)	64.66 (75/116)	20.50 (41/200)	50.00 (109/218)	<0.01
**In the last 6 months**	Number of male sexual partners (mean (SD))	3.47 (6.59)	4.13 (9.15)	2.78 (3.48)	3.85 (7.07)	2.92 (4.09)	<0.01
	Used illegal drugs	7.34 (47/640)	2.50 (5/195)	6.82 (6/88)	13.16 (20/152)	8.00 (16/200)	<0.01
**Transactional sex**		38.11(279/732)	19.50 (39/200)	29.31 (34/116)	62.81 (125/199)	37.33 (81/217)	<0.01
**History of arrested**		13.56 (99/730)	4.00 (8/200)	2.56 (3/117)	12.56 (25/199)	29.44 (63/214)	<0.01
**Living with HIV**		19.57 (144/736)	25.50 (51/200)	19.66 (23/117)	21.39 (43/201)	12.39 (27/218)	<0.01

### Human rights

The proportion of men at all sites who reported experiencing at least one human rights abuse was 46.7% (Table 2). For individual abuses, 6.4% had been denied housing, 5.1% had been denied healthcare, 11.6% had been raped, 10.5% had been beaten by the police, and 18.7% had been blackmailed because of their sexual orientation. Additionally, 16.3% were afraid to walk in their community, and 19.2% were afraid to seek healthcare services.

**Table 2:** Prevalence of human rights abuses reported by MSM in South Africa, Botswana, Malawi, and Namibia.

Human rights abuse or context	Overall % (n/N)	South Africa % (n/N)	Botswana % (n/N)	Malawi % (n/N)	Namibia% (n/N)	p-value (chi2)
Denied housing	6.40 (47/734)	5.00 (10/200)	5.17 (6/116)	6.50 (13/200)	8.26 (18/200)	0.53
Denied healthcare	5.05 (37/733)	5.00 (10/200)	0.85 (1/117)	4.02 (8/199)	8.29 (18/217)	0.02
Blackmailed	18.72 (134/733)	10.50 (21/200)	26.50 (31/117)	18.00 (36/200)	21.30 (46/216)	<0.01
Beaten by the police or a government official	10.50 (77/733)	6.00 (12/200)	1.71 (2/117)	8.04 (16/199)	21.66 (47/217)	<0.01
Raped	11.55 (85/736)	11.00 (22/200)	7.69 (9/117)	11.94 (24/201)	13.76 (30/218)	0.42
Ever experienced any human rights abuse	46.69 (339/726)	41.71 (83/199)	58.62 (68/116)	39.00 (78/200)	52.13 (110/211)	<0.01
Afraid to seek healthcare services	19.18 (141/735)	21.00 (42/200)	20.51 (24/117)	17.50 (35/200)	18.35 (40/218)	0.80
Afraid to walk in community	16.28 (119/731)	9.05 (18/199)	29.06 (34/117)	15.50 (31/200)	16.74 (36/215)	<0.01

In the bivariate analyses comparing men in each study city to Cape Town, no statistically significant difference between locations was found for denial of housing (odds ratio [OR] = 1.04 to 1.71), denial of healthcare (OR = 0.16 to 1.72), or being raped (OR = 0.67 to 1.29) (Table 3). Compared to Cape Town, MSM were over 1.5 times as likely to have been blackmailed in Blantyre and Lilongwe (95% CI 1.05–3.33; p = 0.03), more than twice as likely in Windhoek (95% CI 1.32–4.03; p = 1.32–4.03; p<0.01), and more than three times as likely in Gaborone (95% CI 2.20–7.72; p<0.01). They were also more than 1.5 times as likely to report being afraid to walk in their communities in Blantyre and Lilongwe (95% CI 1.00–3.42; p = 0.05), twice as likely in Windhoek (95% CI 1.11–3.69; p = 0.02), and four times as likely in Gaborone (95% CI 2.20–7.72; p<0.01). Additionally, the likelihood of being beaten by the police was greater in Windhoek (OR = 4.33 [95% CI 2.22–8.44; p<0.01]) than in Cape Town. Finally, MSM in Gaborone and Windhoek were more likely than those in Cape Town to have experienced at least one rights abuse (OR = 1.98 [95% CI 1.24–3.15; p<0.01] and 1.52 [95% CI 1.03–2.25; p = 0.04], respectively).

**Table 3:** Bivariate regression analyses of differences by country in human rights abuses reported by MSM.

Human rights abuse or context	Bivariate (comparing to South Africa)OR (95% CI)*		
	Botswana	Malawi	Namibia
Denied housing	1.04 (0.36–2.93)	1.32 (0.57–3.09)	1.71 (0.77–3.80)
Denied healthcare	0.16 (0.02–1.30)	0.80 (0.31–2.06)	1.72 (0.77–3.82)
Blackmailed	3.07 (1.66–5.66)	1.87 (1.05–3.34)	2.31 (1.32–4.03)
Beaten by the police or a government official	0.27 (0.06–1.24)	1.37 (0.63–2.98)	4.33 (2.22–8.44)
Raped	0.67 (0.30–1.52)	1.10 (0.59–2.03)	1.29 (0.72–2.32)
Ever experienced any human rights abuse	1.98 (1.24–3.15)	0.89 (0.60–1.33)	1.52 (1.03–2.25)
Afraid to seek healthcare services	0.97 (0.55–1.70)	0.80 (0.48–1.31)	0.85 (0.52–1.37)
Afraid to walk in community	4.11 (2.20–7.72)	1.84 (0.99–3.42)	2.20 (1.11–3.69)

*Odds ratios estimate the likelihood of experiencing the rights abuse in each location compared to South Africa (OR>1 indicates more likely than South Africa; OR<1 indicates less likely)

In the multivariate models, only the likelihood of being blackmailed and of being afraid to walk in the community in Gaborone compared to Cape Town remained statistically significant (Table 4). Factors positively associated with being blackmailed included identifying as homosexual or bisexual (Adjusted Odds Ratio [aOR] = 3.11), disclosing sexual orientation (aOR = 1.80), injecting illegal drugs (aOR = 2.41), and engaging in transactional sex (aOR = 2.72). Factors positively associated with ever experiencing any rights abuse included a homosexual or bisexual orientation (aOR = 2.01, p = 0.05), disclosing sexual orientation to family or a healthcare worker (aOR = 1.84, p<0.01), engaging in transactional sex (aOR = 1.5, p = 0.05), history of arrest (aOR = 2.62, p < .01), and being born outside of the study country (aOR = 2.38, p = .03) (Table 5).

**Table 4:** Multivariate regression analyses of differences by country in human rights abuses reported by MSM.

	Multivariate (comparing to South Africa) aOR (95% CI)*							
Country	Human rights abuse or context							
	Denied housing	Denied healthcare	Blackmailed	Beaten by the police or government official	Raped	Ever experienced any human rights abuse	Afraid to seek healthcare services	Afraid to walk in community
**Botswana**	0.76 (0.20–2.83)	0.22 (0.26–1.91)	3.02 (1.43–6.40)	0.57 (0.31–9.90)	0.86 (0.31–2.40)	1.69 (0.96–2.98)	0.91 (0.46–1.79)	2.63 (1.21–5.68)
**Malawi**	0.63 (0.17–2.32)	0.59 (0.13–2.61)	1.16 (0.52–2.58)	1.16 (0.36–3.75)	1.48 (0.61–3.55)	0.74 (0.42–1.31)	0.90 (0.45–1.81)	1.81 (0.79–4.16)
**Namibia**	1.11 (0.39–3.13)	1.81 (0.60–5.46)	1.59 (0.78–3.22)	0.52 (0.19–1.42)	1.54 (0.71–3.36)	1.06(0.64–1.75)	0.73(0.39–1.38)	1.29 (0.59–2.81)

*Each abuse was modeled as an outcome variable.

ORs estimate the likelihood of experiencing each abuse compared to South Africa (OR>1 indicates more likely; OR<1 indicates less likely).

**Table 5:** Multivariate covariates for human rights abuses.

	Human Rights Abuse aOR (95% CI)*								
Descriptive variables		Denied housing	Denied healthcare	Blackmailed	Beaten by the police	Raped	Experienced any rights abuse	Afraid to seek healthcare	Afraid to walk in community
**Age (compared to 18–24 years)**	25–29 years	0.83(0.34–2.01)	1.01 (0.38–2.69)	0.54 (0.30–0.97)	0.82 (0.37–1.82)	0.85 (0.44–1.63)	0.96 (0.63–1.45)	1.30 (0.80–2.12)	0.53 (0.28–1.00)
	30+ years	0.81 (0.27–2.44)	0.68 (0.19–2.42)	0.98 (0.51–1.89)	1.83 (0.77–4.36)	1.06 (0.50–2.24)	0.78 (0.47–1.31)	0.51 (0.24–1.06)	1.12 (0.55–2.28)
**Born in study country**		2.53 (0.77–8.11)	0.75(0.15–3.67)	0.72 (0.30–1.77)	0.15 (0.02–0.94)	0.84 (0.27–2.59)	2.38 (1.11–5.15)	2.21 (1.04–4.72)	1.98 (0.88–4.44)
**Education**	Tertiary/vocational vs secondary or less	0.45 (0.18–1.14)	1.84 (0.71–4.78)	0.93 (0.55–1.57)	0.83 (0.35–1.88)	0.94 (0.51–1.74)	0.79 (0.54–1.17)	1.13 (0.70–1.82)	1.34 (0.77–2.33)
**Employment**	Employed	1.42 (0.65–3.10)	1.10 (0.46–2.63)	0.79 (0.48–1.31)	0.69 (0.35–1.38)	0.77 (0.43–1.37)	1.12 (0.77–1.63)	1.58 (1.00–2.51)	1.38 (0.81–2.35)
**Sexual orientation**	Homosexual or bisexual vs heterosexual	2.24 (0.75–6.65)	7.17 (2.28–22.58)	3.11 (1.44–6.72)	2.31 (0.96–5.55)	3.58 (1.60–8.03)	2.01 (1.01–3.99)	3.20 (1.53–6.71)	1.88 (0.82–4.44)
**Disclosed sexual orientation**		2.40 1.02–5.65)	6.30 (2.03–19.53)	1.80 (1.07–3.06)	0.90 (0.44–1.82)	2.08 (1.11–3.92)	1.84 (1.24–2.75)	2.22 (1.34–3.69)	1.56 (0.90–2.70)
**In the last 6 months**	Four or more male partners	1.51 (0.61–3.71)	0.94 (0.33–2.70)	1.67 (0.92–3.02)	0.67 (0.28–1.62)	1.60 (0.81–3.16)	1.15 (0.69–1.91)	1.04(0.57–1.89)	1.04(0.51–2.11)
	Injected illegal drugs	1.95 (0.66–5.81)	4.67 (1.34–16.25)	2.41 (1.11–5.23)	2.75 (1.00–7.62)	2.31 (0.94–5.68)	1.95 (0.90–4.20)	2.22 (1.04–4.76)	2.53 (1.13–5.66)
**Transactional sex**		1.94 (0.87–4.39)	1.68 (0.66–4.29)	2.72 (1.63–4.55)	3.72 (1.83–7.55)	1.15 (0.62–2.14)	1.50 (1.00–2.25)	1.23 (0.75–2.02)	1.06 (0.60–1.87)
**History of arrest**		1.38(0.55–3.49)	1.44 (0.52–4.01)	1.36 (0.72–2.59)	9.48 (4.74–18.98)	0.83(0.38–1.81)	2.62 (1.50–4.60)	1.30 (0.69–2.44)	2.47 (1.27–4.78)
**Living with HIV**		1.21 (0.48–3.05)	2.60 (1.01–6.72)	0.96 (0.52–1.77)	0.75 (0.32–1.80)	1.85 (0.97–3.50)	1.12 (0.71–1.76)	0.89 (0.75–2.02)	0.59 (0.29–1.23)

*Odds ratios estimate the association between each abuse and descriptive variable.

OR>1 indicates respondents with the characteristic are more likely to experience the abuse. OR<1 indicates these respondents are less likely to experience the abuse.

## Discussion

This study serves as an exploration of the circumstances in which MSM in Southern African countries with different legislative contexts live. Nearly half of all participants, including those in South Africa with its supportive legislation, reported at least one human rights abuse in their lifetime, demonstrating the vulnerability, stigma, and discrimination these men face. As Reid and Dirsuweit argue, this may be because the South African constitution allows for increased visibility of homosexuality that is seen to subvert the traditionally heterosexual landscape and these abuses are a response to this subversion. [37] Sexual orientation and disclosure of orientation were positively correlated with experiencing a human rights abuse, supporting earlier studies showing MSM who identify as gay are more vulnerable than their heterosexual counterparts and remain in acute need of security. [38]

A high prevalence of blackmail was reported in this study, including by 10.5% of Cape Town participants. Still, in the bivariate analyses, this was statistically significantly lower than participants in other study sites, and remained lower than rates in Gaborone in the multivariate analysis. Similarly, MSM in Cape Town were less afraid to walk in their communities. It may be that in Botswana, Malawi, and Namibia, blackmailers can leverage anti-homosexuality laws knowing their victims will not report abuse or blackmail for fear of being persecuted themselves. The more favourable South African political atmosphere may have created an environment in which MSM can be open without fear. Interestingly, MSM in Gaborone reported the highest rates of fear despite reporting the lowest rates of many of the abuses explored in this study. Since MSM often experience verbal or other abuses not assessed here, MSM in Botswana may more often be victims of these abuses, possibly explaining the elevated rates of fear. [4, 5] These high rates of abuse particularly support the need for community-level interventions and grassroots action addressing stigma and discrimination locally. [39] Conscious efforts must be made to support local gay cultures and legitimize minority sexual identities. [15] This may prove difficult in countries with discriminatory laws, but support of local gay responses and public education will go far to normalize same-sex sexualities and address social dimensions of homophobia.

There was a strong correlation between disclosure of sexual orientation and denial of healthcare, and MSM in Cape Town were equally likely to be afraid to seek healthcare as MSM in other study cities. Fear of accessing care was again particularly high in Botswana, despite high rates of disclosure to healthcare workers and little denial of care. This may be due to negative experiences in the health system other than denial of care, as earlier studies have reported discrimination from healthcare providers. [31, 40] In such settings, MSM are less likely to openly discuss their sexuality with providers and more likely to provide incomplete or inaccurate sexual histories. [41] As providers who are fully aware of the sexual practices of their patients are better able to provide appropriate services, providers must respect their obligation to provide services free from discrimination.

Additionally, as the South African legal framework is supportive of all sexual orientations, the enabling environment exists to increase provider competencies regarding these groups. MSM sensitivity training is one way to improve attitudes concerning same-sex sexualities and equip providers to offer accessible and informed services. These results suggest further work is needed to train larger cohorts to support lasting change, as trained providers returning to their workplace often report little support from untrained colleagues. [42] This competency-building should be incorporated into systematic education of providers in all countries.

To fully address issues facing MSM, a comprehensive approach with interventions, leadership, and activism at multiple levels in multiple sectors is needed. The South African legal system has attempted to ensure the universality of human rights, but legal action alone is insufficient to prevent MSM from experiencing abuse. Perceptions exist in South Africa, along with other study countries, that being homosexual or being labelled one is degrading. [40] Change in social attitudes must accompany legal change to reduce rates of abuse experienced by these men.

## Conclusion

These findings highlight the high levels of human rights abuses experienced by MSM in Southern Africa. However, decreased rates of blackmail and fear reported in Cape Town suggest the South African legal framework may positively impact the lives of these men. While this provides evidence that decriminalization of same-sex behaviour is a critical first step, it must be accompanied by comprehensive efforts to normalize same-sex behaviour, enforcement of laws providing protection from discrimination, and systematic sensitization of the people to dislodge prejudices, including in cultural and religious contexts. Even where policy includes people in same-sex relationships and protection of human rights as guiding principles, protecting MSM from abuse is made more difficult by the lack of government commitment and negative public opinion. These data suggest both legal and social change is needed to change attitudes regarding sexual minorities and address discrimination and violence affecting MSM.

## Supporting Information

S1 File MSM Survey Instrument. (DOC)
