# Supplementary material for: Human Rights Violations among Men Who Have Sex with Men in Southern Africa: Comparisons between Legal Contexts
Source: PLoS One. 2016 Jan 14;11(1):e0147156. doi: 10.1371/journal.pone.0147156 (PMC4713169; doi:10.1371/journal.pone.0147156)
Supplement: S1 File — (DOC) [file pone.0147156.s001.doc]

S1: Survey Instrument

| Country |  | City |  | | Result | |
| --- | --- | --- | --- | --- | --- | --- |
| Participant Code |  | Date |  | |
| **Question Number** | **Question** | | | **Response Guide**  88 Unsure/don’t know  99 No response | | **Response**  (Numerical Answer) |
| 1 | How old are you? | | | Number of Years | |  |
| 2 | Were you born in this country? | | | 1 yes  2 no | |  |
| 3 | Did you grow up in a rural area? | | | 1 yes  2 no | |  |
| 4 | What level of education have you attained? | | | 1 No formal Education  2 Primary School  3 Secondary School  4 Tertiary or Vocational School | |  |
| 5 | Are you currently employed/self-employed? | | | 1 yes  2 no | |  |
| 6 | Do you currently have a boyfriend? | | | 1 yes  2 no | |  |
| 7 | Are you either married to or in a relationship with a woman? | | | 1 yes  2 no | |  |
| 8 | Which of the following terms best describes your sexual orientation? | | | 1 Heterosexual/Straight  2 Homosexual/Gay  3 Bisexual  4 Transgender  5 Other (please specify) | |  |
| 9 | Have you told your immediate family that you have sex with other men? | | | 1 yes  2 no | |  |
| 10 | Have you told anyone in your extended family that you have sex with other men? | | | 1 yes  2 no | |  |
| 11 | Have you told any medical personnel at a hospital or clinic that you have sex with other men? | | | 1 yes  2 no | |  |
| 12 | Have you ever used the internet with a computer or mobile phone to find a male partner? | | | 1 yes  2 no | |  |
| 13 | Which of the following do you currently consider to be the biggest threat to your health? (Choose only one answer) | | | 1 Violence  2 Malaria/Tuberculosis  3 HIV/AIDS  4 Other Sexually Transmitted Infections (STI)  5 Diabetes/Heart Disease/Cancer  6 Mental Health  7 Illegal Drugs  8 Other | |  |
| 14 | Can you get HIV from anal sex with a man? | | | 1 yes  2 no | |  |
| 15 | Can you get HIV from sex with a woman? | | | 1 yes  2 no | |  |
| 16 | Can you get HIV from using a needle to inject illegal drugs? | | | 1 yes  2 no | |  |
| 17 | Have you ever received information about how to prevent getting HIV from women? | | | 1 yes  2 no | |  |
| 18 | Have you ever received information about how to prevent getting HIV from men? | | | 1 yes  2 no | |  |
| 19 | Approximately how many male sexual partners have you had in the last 6 months? | | | Number of Partners | |  |
| 20 | Approximately how many female sexual partners have you had in the last 6 months? | | | Number of Partners | |  |
| 21 | Approximately how many times have you injected illegal drugs in the last 6 months? | | | Number of Times | |  |
| 22 | How often do you use a condom with your boyfriend? | | | 1 always  2 sometimes  3 rarely  4 never  77 No boyfriend | |  |
| 23 | How often do you use a condom for anal sex with *casual* male partners? | | | 1 always  2 sometimes  4 rarely  5 never  77 No casual male partners | |  |

| **Question Number** | **Question** | **Response Guide**  88 for Unsure/don’t know  99 for No response | **Response**  (Numerical Response) |
| --- | --- | --- | --- |
| 24 | Which type of lubricant do you use with condoms for anal sex with men? | 1 petroleum jelly or Vaseline  2 body creams/fatty creams  3 water-based lubricant  4 saliva  5 uses condoms, but no lubricant  77 No condom use |  |
| 25 | How often do you use a condom with your girlfriend or wife? | 1 always  2 sometimes  3 rarely  4 never  77 No girlfriend or wife |  |
| 26 | How often do you use a condom with *casual* female partners? | 1 always  2 sometimes  3 rarely  4 never  77 No casual female partners |  |
| 27 | Has a clinic worker or other health professional ever told you that you had a sexually transmitted infection? | 1 yes  2 no |  |
| 28 | Has a health professional ever treated you for a STI? | 1 yes  2 no |  |
| 29 | Has a health professional ever recommended that you get an HIV test? | 1 yes  2 no |  |
| 30 | If ever, when was the last time that you were tested for HIV? | Number of **Months** Since Test  77 Never tested |  |
| 31 | Has a clinic worker or other health professional ever told you that you were infected with HIV or that you have AIDS? | 1 yes  2 no |  |
| 32 | Are you currently being treated with medications for HIV, including cotrimoxazole (CTX)? | 1 yes  2 no |  |
| 33 | Have you ever been denied housing for any reason other than not being able to pay? | 1 yes  2 no |  |
| 34 | Have you ever been denied health care services on the basis of your sexuality? | 1 yes  2 no |  |
| 35 | Have you ever been afraid to seek health care services? | 1 yes  2 no |  |
| 36 | Have you ever received money or gifts to have casual sex with a man? | 1 yes  2 no |  |
| 37 | Have you ever paid money or gifts to have casual sex with a man? | 1 yes  2 no |  |
| 38 | Have you ever been blackmailed because of your sexuality? | 1 yes  2 no |  |
| 39 | Have you ever been arrested? | 1 yes  2 no |  |
| 40 | If you have ever been to jail, did you have access to condoms while in prison? | 1 yes  2 no  77 Not been to prison |  |
| 41 | Are you afraid to walk down the street in your community? | 1 yes  2 no |  |
| 42 | Have you ever been beaten up by the police or a government official? | 1 yes  2 no |  |
| 43 | If you have been beaten up in the last 12 months, who beat you up? (Can choose more than one answer) | 1 Family member  2 Classmate  3 Sexual Partner  4 Police/Government Official  6 Unrelated male in community  7 Housemate/Roommate  8 Fellow worker  77 Not been beat up |  |
| 44 | If you have ever been raped, did you report it? | 1 yes  2 no  77 Never been raped |  |
| 45 | If you have been raped, was it prosecuted in a court of law? | 1 yes  2 no  77 Never been raped |  |
